# Five Advances for Benign Foregut Surgery in the Last 50 Years

**DOI:** 10.1002/wjs.70400

**Published:** 2026-05-06

**Authors:** David I. Watson, Sarah K. Thompson, John G. Hunter, B. Mark Smithers

**Affiliations:** ^1^ Flinders Health and Medical Research Institute and College of Medicine and Public Health Flinders University Adelaide South Australia Australia; ^2^ Department of Surgery Flinders Medical Centre Adelaide South Australia Australia; ^3^ Adelaide Gastrointestinal Specialists Eastwood South Australia Australia; ^4^ Division of Foregut and Bariatric Surgery Department of Surgery Oregon Health & Science University School of Medicine Portland Oregon USA; ^5^ Division of Surgery Princess Alexandra Hospital Brisbane Australia; ^6^ Faculty of Health, Medicine and Behavioural Sciences University of Queensland Brisbane Queensland Australia

Foregut surgery began in 1881 when Theodor Bilroth “successfully” performed the first distal gastrectomy for a 43 year‐old woman with gastric cancer [1]. Even though she died from metastatic cancer 4 months later, this was deemed a “success” as all previous attempts had failed due to early postoperative deaths. Bilroth's advance was only possible following the development of anesthesia and advances in antisepsis. His work opened a new era of abdominal surgery where “big” operations were shown to be possible. In the area of foregut surgery, gastrectomy, pyloroplasty, and vagotomy were applied to the problem of peptic ulcer, and surgery for gastroesophageal reflux and hiatus hernia was undertaken via open thoracic and abdominal approaches. Three of the authors trained in general surgery at the end of that era. At that time, open surgery for bleeding peptic ulcer was one of the commonest operations performed by general surgeons when on‐call.

However, since the late 1980's foregut surgery has changed significantly. Peptic ulcer disease now rarely troubles surgeons, and open incisions to access the abdomen and chest have now been largely replaced by minimal access approaches. Further, problems such as obesity that weren't considered to be “surgical” became a surgical problem, even though the advent of GLP‐1 inhibitors might limit the future use of surgery for obesity. What is clear is that over the last 50 years, the diseases presenting to foregut surgeons and the types of procedures they perform have changed dramatically. Surgeons from an earlier era might struggle to recognize current practice. Whilst there have been many changes, we propose five advances that have each changed the field of foregut surgery.

## 1 A Shift From Open to Minimally Invasive Surgery

Following the development of small cameras attached to laparoscopes displaying the abdominal cavity on a video screen, as well as instruments for intervention via laparoscopic ports, the first laparoscopic cholecystectomies were performed in the late 1980's [2]. This paved the way to the current era of minimal access surgery where laparoscopic approaches are now applied for the vast majority of benign foregut surgery. Minimal access foregut surgery began in 1991 with the first descriptions of laparoscopic approaches for Nissen fundoplication. Geagea and Dallemagne et al. reported small case series, which were followed by a rapid shift to laparoscopic approaches for antireflux surgery [3, 4]. Within a few years large series were reported, confirming the feasibility and safety of these approaches [5, 6]. Subsequent randomized trials provided level I evidence of better outcomes following laparoscopic Nissen fundoplication than open Nissen fundoplication, with excellent reflux control, shorter hospital stays and less complications demonstrated [7, 8].

Techniques for laparoscopic repair of large hiatus hernia, revisional antireflux surgery, cardiomyotomy for achalasia, and excision of benign gastric tumors such as gastrointestinal stromal tumors were all described in the 1990's. Outcomes following laparoscopic approaches are now well understood and deliver lower complication rates and shorter hospital stays. Consequently, almost all benign foregut surgery is now undertaken via minimal access approaches, with the more recent robotic systems now pushing the debate toward what type of type of minimal access approach, laparoscopic versus robotic, is most likely to be used across the next decade. For now, conventional laparoscopy holds sway for benign foregut surgery as techniques are reliable and well understood, and conventional instrumentation is cheaper and more readily available than robotic platforms.

## 2 The Evolution and Refinement of Fundoplication

By the 1980's a consensus had been reached across much of the surgical world that the best operation for gastroesophageal reflux was a Nissen fundoplication. Originally described as a 5 cm long 360‐degree wrap of the gastric fundus around the distal esophagus via an open surgical approach, case‐control and cohort studies suggested better outcomes if the fundoplication was short (1–2 cm in length) and constructed in a loose fashion after dividing the short gastric blood vessels [9, 10]. Even though this approach improved on Nissen's original procedure, it was followed by troublesome side effects in some individuals [5]. The shift to laparoscopic approaches was associated with a concentration of the surgical case load from generalists to subspecialist foregut surgeons in much of the developed world, providing these surgeons the opportunity to rapidly accumulate large experiences, and report short and eventually longer‐term outcomes. Outcomes from large laparoscopic cohort studies highlighted problems with persistent dysphagia, flatulence and bloating, which required permanent dietary modification in some patients [5]. These issues encouraged the evaluation of alternative approaches, such as partial fundoplication, which offered reflux control with potentially less side effects [11, 12].

The large case‐loads in many centers offered a unique opportunity to evaluate alternative approaches with randomized controlled trials so that, across the late 1990's and early 2000's, randomized trials were reported comparing fundoplication versus medical therapy for gastroesophageal reflux, laparoscopic versus open fundoplication, Nissen fundoplication with versus without short gastric vessel division, and Nissen versus posterior or anterior partial fundoplication [13]. Meta‐analyses and network meta‐analyses have now shown that fundoplication achieves better reflux control than medical therapy, a laparoscopic approach to surgery is preferred, division of short gastric vessels for Nissen fundoplication does not improve outcome, and partial fundoplications are followed by less side effects than Nissen fundoplication but perhaps offset by a higher risk of recurrent reflux [14]. Underpinned by evidence from randomized trials, fundoplication in many centers has now evolved away from the approach advocated in the 1980's to approaches that Nissen might now struggle to recognize, with anterior or posterior partial fundoplications now preferred in many centers as they offer effective reflux control with fewer side effects [11, 12].

## 3 The Decline and Fall of Surgery for Peptic Ulcer!

When three of the authors trained in general surgery, it was widely believed that peptic ulcers were caused by acid, aggravated by stress. At that time widely available acid suppressing medication was limited to histamine type 2 receptor antagonists, and proton pump inhibitors were undergoing clinical trials. Elective gastric resection, a once revered part of the general surgeon's operative repertoire, was being replaced by highly selective vagotomy for non‐healing duodenal ulcers, and bleeding peptic ulcers were considered to be a surgical problem which required treatment with either a distal partial gastrectomy for a gastric ulcer or underrunning with sutures for a duodenal ulcer, with emergency procedures generally combined with truncal vagotomy.

Marshall and Warren's discovery of *Helicobacter pylori* as the major cause of peptic ulcer disease led to a paradigm shift in the management of peptic ulcer disease, from a surgical disease to a problem that is now cured by a course of antibiotics [15]. Recognition of the significance of *Helicobacter pylori* led to the demise of elective surgery for peptic ulcer disease by the early 1990's, and a more gradual decline in emergency surgery for bleeding peptic ulcer, such that over the last 20 years emergency surgery for peptic ulcer diseases in the developed world is now largely limited to perforated ulcers due to excessive use of nonsteroidal anti‐inflammatory agents. Surgery for a bleeding peptic ulcer is now a rare event, driven by less frequent presentations, and the concurrent development of effective interventional endoscopy techniques which are highlighted in the next section.

## 4 Advances in Diagnostic and Interventional Gastroscopy

Gastroscopy using a flexible fiberoptic endoscope was initially developed in 1957 by Hirschowitz and colleagues in Michigan, USA [16], and then refined and further developed in Japan. By the 1980's, diagnostic endoscopy was widely applied by gastroenterologists as well as general surgeons in some countries. Initially this revolutionized the diagnosis of foregut disease. Further refinement of endoscope design facilitated interventional approaches such as cautery, sclerotherapy and clipping of bleeding vessels within peptic ulcers. These developments drove a shift from surgery to interventional endoscopy for the management of bleeding from the stomach and duodenum, such that surgery is now rarely required. Further refinement of endoscopes from fiberoptic to video endoscopes with improved image resolution has allowed the identification and endoscopic treatment of mucosal neoplasia. Over the last two decades new instrumentation has allowed submucosal dissection within the wall of the esophagus and stomach to facilitate the removal of neoplastic and preneoplastic mucosal lesions, as well as new approaches such as peroral endoscopic myotomy (POEM) for the treatment of achalasia and, in highly selected cases, G‐POEM for pyloric myotomy [17, 18]. What is clear from this development is that the boundaries between surgery and endoscopy are now blurred, with flexible endoscopy established as a major tool for diagnosis and treatment of foregut disease.

## 5 Surgery for Obesity—A Rapid Rise, and Potential Decline

Before the 1990's open surgery for obesity was performed for some individuals with severe obesity. Given patient size and access required it was associated with significant morbidity, so that these procedures were generally only considered for a small number of morbidly obese individuals. The development of laparoscopic approaches in the 1990's shifted opinion toward the more liberal use of surgery. Laparoscopic gastric bypass was described in 1994 [19], but was not widely adopted at that time due to the complexity of the procedure which entailed gastric division and anastomoses. Surgeons undertaking these procedures laparoscopically had to have a high level of technical proficiency.

An alternative approach, the laparoscopic adjustable gastric band (LAGB), was described in 1993 by Belachew in Belgium [20]. As this offered a technically simpler procedure which was within the skill set of most general surgeons, LAGB took off and surgery rates for obesity increased exponentially in many parts of the world. For example, in Australia the annual rate of surgery for obesity increased 50‐fold from 1994 to 2006 [21]. Unfortunately, this was followed by a similar increase in the rate of revision and reversal surgery, and this led surgeons to eventually abandon the LAGB procedure. Despite evidence from randomized trials showing that gastric bypass achieved better weight loss and lower rates of surgical revision than procedures that rely entirely on gastric restriction [22], the decline in LAGB was followed by an similar rapid increase in the application of an alternative restrictive procedure, laparoscopic sleeve gastrectomy, a procedure which is also followed by a significant risk of revision surgery, often due to difficult refractory gastroesophageal reflux [23].

What is clear is that there have been cycles of enthusiasm when surgeons perceive an easy to perform operation for morbid obesity, followed by disappointment when longer term outcome data shows unacceptable rates of failure. For surgery to progress in this area, new developments require better evaluation, ideally within well designed randomized trials, before wide dissemination.

More recently, the whole area of surgery for obesity has come under threat from medical therapies. The development of GLP‐1 inhibitors, and randomized trials and cohort studies showing that similar short to medium term weight loss outcomes can be achieved by medical therapy, is likely shifting treatment demand from surgery to medical therapy [24]. The final impact of this shift remains to be seen, but it is likely to significantly impact the number of people seeking a surgical solution for obesity, with surgery likely to have a diminished role in future practice.

## 6 Conclusion

Over the past 50 years foregut surgery has evolved considerably, with a shift to minimally invasive procedures driven by better laparoscopic equipment and improved safety and side‐effect profiles. We have witnessed the eradication of one of the most commonly performed procedures in the general surgeon's repertoire (gastrectomy for peptic ulcer disease), incredible advances in our understanding and management of hiatus hernia and gastroesophageal reflux disease, the rise (and fall) of bariatric procedures, and rapid uptake of interventional endoscopic techniques to treat bleeding ulcers, early cancers, and motility disorders.

There is no doubt that we will see a different pattern of foregut surgery in a further 50 years. We predict that minimally invasive surgery to repair hiatus hernia and reflux will stand the test of time, but bariatric surgery will probably disappear, as has surgical interventions for peptic ulcer disease. Inevitably, there will be newer and better endoscopic and robotic platforms to the point that surgeons may no longer need to scrub to perform complex operations! We look forward to ongoing advances over the next 50 years.

## Author Contributions

**David I. Watson:** conceptualization, writing – original draft, writing – review and editing. **Sarah K. Thompson:** writing – review and editing. **John G. Hunter:** writing – review and editing, conceptualization. **B. Mark Smithers:** writing – review and editing, conceptualization.

## Funding

The authors have nothing to report.

## Conflicts of Interest

The authors declare no conflicts of interest.

## Data Availability

Data sharing not applicable to this article as no datasets were generated or analysed during the current study.
